# Tailoring acidity of HZSM-5 nanoparticles for methyl bromide dehydrobromination by Al and Mg incorporation

**DOI:** 10.1186/1556-276X-9-550

**Published:** 2014-10-03

**Authors:** Zhen Liu, Zhongdong Zhang, Wei Xing, Sridhar Komarneni, Zifeng Yan, Xionghou Gao, Xiaoping Zhou

**Affiliations:** 1 State Key Laboratory of Heavy Oil Processing; Key Laboratory of Catalysis, CNPC, China University of Petroleum, Qingdao 266580, People's Republic of China; 2 Department of Chemical Engineering, Hunan University, Changsha 410082, People's Republic of China; 3 Lanzhou Petrochemical Research Center, Petrochemical Research Institute, PetroChina Company Limited, Lanzhou 730060, People's Republic of China; 4 Materials Research Institute, The Pennsylvania State University, University Park, PA 16802, USA

**Keywords:** HZSM-5 nanoparticles, Acidity tailoring, Methyl bromide, Dehydrobromination

## Abstract

Three kinds of HZSM-5 nanoparticles with different acidity were tailored by impregnating MgO or varying Si/Al ratios. Both the textural and acidic properties of the as-prepared nanoparticles were characterized by nitrogen adsorption-desorption measurements, X-ray diffraction (XRD), scanning electron microscopy (SEM), ammonia temperature-programmed desorption (NH_3_-TPD) and Fourier transform infrared spectroscopy (FTIR or Py-FTIR). It was found that the intensity of Lewis acid sites with weak strength was enhanced by impregnating MgO or reducing Al concentration, and such an enhancement could be explained by the formation of Mg(OH)^+^ or charge unbalance of the MgO framework on the surface of HZSM-5 support. The effect of HZSM-5 nanoparticles' acidity on methyl bromide dehydrobromination as catalyst was evaluated. As the results, MgHZ-360 catalyst with the highest concentration of Lewis acid sites showed excellent stability, which maintained methyl bromide conversion of up 97% in a period of 400 h on stream. Coke characterization by BET measurements and TGA/DTA and GC/MS analysis revealed that polymethylated naphthalenes species were formed outside the channels of the catalyst with higher acid intensity and higher Brønsted acid concentration during the initial period of reaction, while graphitic carbon formed in the channels of catalyst with lower acid intensity and higher Lewis acid concentration during the stable stage.

## Background

Currently, natural gas is industrially converted to syngas by steam reforming reaction, which then can be converted either to liquid fuel by Fisher-Tropsch (F-T) process directly [1] or to methanol using Cu/ZnO/Al_2_O_3_ catalyst [2]. Methanol can also be converted into higher hydrocarbons catalyzed by acidic zeolite, which is commonly referred to as methanol-to-hydrocarbon (MTH) reaction. The MTH reaction could be altered with different process conditions or catalyst choice to transform methanol to gasoline (MTG) [3] or methanol to olefins (MTO) [4]. Through the MTH technology, one could make almost any fuels or chemicals from methanol that can currently be made from crude oil.

Although F-T or MTH process could convert natural gas into liquid fuel as alternative to crude oil, an unavoidable reality is that syngas should be firstly synthesized. Normally, syngas is produced by steam reforming reaction, which is a highly energy-cost, scale-sensitive and water-needed process [5]. The proven reserves of natural gas have doubled in the last decade, unfortunately, mainly from increases in ‘unconventional’ gas found with shale (shale gas), coal (coal bed methane), and in low-permeability ‘tight’ sandstones (tight gas) [6]. Furthermore, the location of such unconventional gas is usually far away from abundant water sources and inconveniently transported. All those reality of the condition limits cost-effective utilization of natural gas via syngas process on a world-wide scale [7].

**Figure 1:**
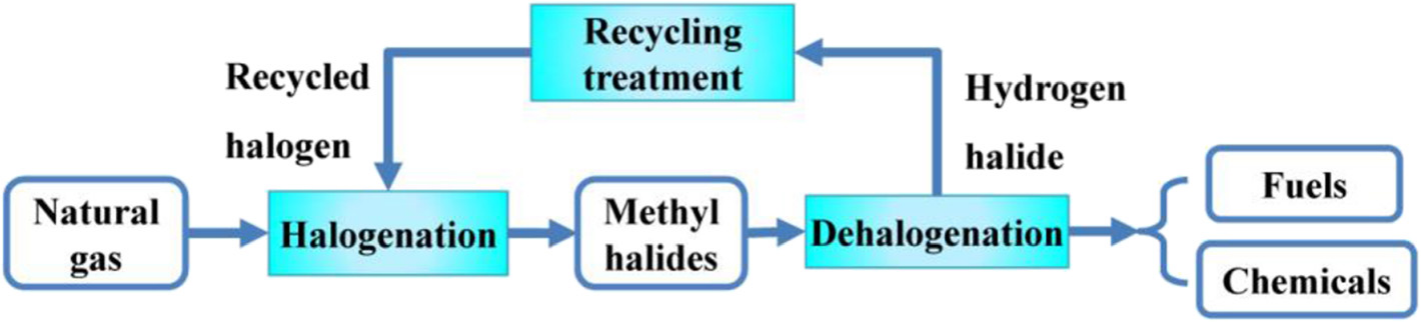
Illustration of halide-mediated process for converting natural gas.

Considering the similarity of molecular structure between methanol and methyl halide (CH_3_Cl or CH_3_Br), some also mentioned the possibility of converting methyl halides to hydrocarbons [8]. A novel halide-mediated process was proposed to produce liquid fuels or chemicals, which can illustrate as Figure 1. In this process, methane, the major component of natural gas, is firstly halogenated to methyl halide, and methyl halide could be, in turn, converted into liquid fuels or chemicals. The released HBr (if using methyl bromide as intermediate) during the second step could be regenerated by a recycling treatment for reuse. Although HBr is relatively expensive and a potent greenhouse gas, it is only recycled in the process for good and minimally harm the environment. Compare with syngas-mediated strategy, methyl halide-mediated route shows unique advantages, such as lower operating pressure and temperature, water-free reaction, and mild exothermic property.

Methyl halide conversion to hydrocarbons has been investigated since the mid-1980s. The product distribution of methyl halide to hydrocarbons is strikingly similar to methanol conversion over the same topology [9]. The previous reports in literatures are mainly focused on the conversion of methyl chloride over various zeolite catalysts [8,10-15]. Considering the energy of X-C bond (X stands for halide atoms), methyl bromide has significant advantages over methyl chloride due to the weaker Br-C bond relative to Cl-C, which means that the Br-C bond is easily dissociated than the Cl-C bond under mild condition [6]. However, the synthesis of higher hydrocarbons utilizing methyl bromide as a reactant is occasionally mentioned. Olsbye et al. [16] investigated both methyl chloride and methyl bromide as reactants for dehydrohalition over H-SAPO-34 catalyst. However, the catalyst endured a harsh deactivation situation due to the formation of severe carbonaceous deposit.

HZSM-5 is known as an efficient catalyst for the MTH process. It has always been very tricky to explain the mechanism of formation of the first carbon-carbon bond in MTH reaction. Currently, beliefs are steadily shifting toward the so-called hydrocarbon pool mechanism, which was suggested by Dahl and Kolboe [17,18]. The hydrocarbon pool, with an initially specified overall stoichiometry (CH_2_)_*n*_, was said to represent an adsorbate in the channels of catalyst and acted like a ‘matrix.’ Methanol reactant is continuously added to an adsorbate followed by the splitting of light olefins via subsequent dealkylation of the adsorbate. Light olefins splitting from the carbon pool can undergo further rapid secondary reactions, such as hydrogen transfer reaction, alkylation, isomerization, and cracking reaction, which finally lead to complex higher hydrocarbon distribution. Olsbye et al. [19] revealed that HZSM-5 can also be used as catalyst for the conversion of CH_3_Cl into C_2_-C_4_ paraffins and C_5_^+^ aromatics. Wang et al. [20,21] revealed a fluoride-treated H-ZSM-5 as a highly selective and stable catalyst for the propylene production from methyl halides. In our previous work, value-added chemicals such as dimethyl ether, higher hydrocarbons, and acetic acid were successfully synthesized from methyl bromide [22-25]. For the production of higher hydrocarbon [23], a catalyst of MgO/HZSM-5 was presented without further data in detail on catalytic performances, especially on the effect of acid property on catalyst activity and lifetime. Herein, three HZSM-5 samples with different acidities tailored by changing Si/Al ratios or impregnating certain amount of MgO are evaluated. The influence of the catalyst's acid strength and intensity on catalytic methyl bromide dehydrobromination performance and resistance to deactivation was also comprehensively investigated.

## Methods

### Catalyst preparation

MgO/HZSM-5 catalysts were prepared by impregnating commercially available HZSM-5 (9.80 g, with respective Si/Al ratio of 360, 100, and 50) with 2.0 wt.% of MgO using an aqueous solution of Mg(NO_3_)_2_ ⋅ 6H_2_O (1.28 g dissolved in 30.0 ml H_2_O) as precursor. After stirring for 2 h, the mixture was dehydrated at 80°C in a water bath for approximately 4 h and dried at 120°C for another 2 h in oven. Then, all the samples were calcined at 450°C for 8 h to obtain the final catalysts. The catalysts were named as MgHZ-360, MgHZ-100, and MgHZ-50, respectively, in which the numbers 360, 100, and 50 stand for Si/Al ratios of the corresponding HZSM-5 zeolite. The catalysts were pressed, crushed, and sieved to 20 to 60 mesh before using.

### Catalyst characterization

The X-ray diffraction (XRD) characterization of the catalysts was performed using Philips PW3040/60 X-ray diffractometer with Cu Kα radiation (Philips, Amsterdam, The Netherlands). The measurements were run in step-scan mode with steps of 0.02° in the range of 5° to 60°. Brunauer, Emmett and Teller (BET) surface area measurement of the samples was performed using a Beckman Coulter SA 3100 adsorption instrument (Beckman Coulter Inc., Brea, CA, USA) with N_2_ as adsorbent after degassing the samples at 250°C for 2 h. Scanning electron microscope (SEM) micrographs were taken on a JEOL JSM 6390 instrument (JEOL Ltd., Tokyo, Japan).

Temperature-programmed desorption of ammonia (NH_3_-TPD) instrument (Micromeritics Auto Chem II 2920; Micromeritics Instrument Corporation, Norcross, GA, USA) was employed to characterize the acidity of the catalysts. In each NH_3_-TPD measurement, *ca*. 200 mg of the catalyst was loaded into a quartz microreactor. The catalyst was pretreated by heating the sample from room temperature to 650°C at a rate of 15°C/min in helium with flow rate of 50.0 ml/min, then the microreactor was cooled down and maintained at 150°C before NH_3_ adsorption. Ammonia adsorption was carried out at 150°C by switching argon to helium-ammonia mixture (10.0 vol.% NH_3_) with a flow rate of 30.0 ml/min. After 30 min of ammonia adsorption, the sample was flushed with pure helium (30 ml/min) for 30 min to remove the gaseous NH_3_. In the desorption process, the temperature was increased from 150°C to 600°C at a rate of 15°C/min in carrier gas helium (50.0 ml/min), and the desorbed NH_3_ was analyzed by an online thermal conductivity detector.

Fourier transform infrared spectroscopy (FTIR) spectra of samples before and after pyridine adsorption (Py-FTIR) were collected on a Nexus 670 FTIR equipment (Thermo Nicolet; Thermo Fisher Scientific Hudson, NH, USA) with a high-vacuum reaction cell (HVC) from Harrick Science (Harrick Scientific Products, Inc., NY, USA). A KBr beam splitter and two CaF_2_ windows were employed. Each spectrum was collected with 32 scans at 4 cm^-1^ resolution. For FTIR investigation, approximately 200 mg of the sample was weighed and pelletized before putting into the sample cell. Before spectrum collection, the sample was first thermally pretreated at 350°C for 1 h under high-purity N_2_, then another 1 h under high-vacuum condition. After the thermal treatment, the cell was cooled down to 100°C for spectrum collection. For pyridine adsorption determination after cooling down, the spectrum was collected as a background before the pyridine adsorption step. Then, N_2_ valve was switched to a pyridine bubbler (maintained at 30°C) to saturate a certain amount of pyridine. The mixture of N_2_ and pyridine was passed though the sample cell for 30 min of adsorption. After purging the cell with high-purity N_2_ for another 30 min, a spectrum at 100°C was acquired.

The amount of coke deposited on the spent catalyst was determined by thermogravimetric analysis/differential thermal analysis (TGA/DTA) method under air atmosphere (Netzsch STA449C; Erich Netzsch GmbH & Co. Holding KG, Selb, Germany). The spent catalyst after deactivation or predetermined time of reaction was loaded in the crucible of the microbalance followed by heating from room temperature to 800°C at the rate of 10°C/min. The weight loss and differential thermal analysis curves were recorded.

The analysis of compounds trapped in the spent catalyst was performed according to the method described in the previous literature [26]. In each analysis, 200 mg of used catalyst was dissolved in 1.0 ml of 15% HF solution in a Teflon vessel. After catalyst was dissolved in HF solution, 1 M of NaOH solution was added to neutralize the acid. The resulting solution was extracted by 1.0 ml CCl_4_. The organic extract was analyzed on a gas chromatograph/mass spectrometer (GC/MS, Agilent 6890 N/5973 N; Agilent Technologies, Sta. Clara, CA, USA) with an HP-5MS column (30 m × 250 μm × 0.25 μm).

### Catalytic testing

The CH_3_Br dehydrobromination was carried out in the reactor (ID 1.5 cm, length 35.0 cm) containing 8.0 g of catalyst with both ends filled with quartz sands (20 to 40 mesh) at the reaction temperature of 280°C. Typically, the reactions were carried out with 18.0 ml/min of CH_3_Br and 5.0 ml/min of N_2_ used as an internal standard. The liquid products and the gas effluent were analyzed by a GC with thermal conductivity detector (Agilent 6890 N; Agilent Technologies, Sta. Clara, CA, USA) and a GC/MS (6890 N/5973 N). The conversion of CH_3_Br was calculated by referencing to the internal standard N_2_ by the Equation 1:

(1) $$X_{MB}\%=\frac{{A_{1}}^{\prime }/{A_{0}}^{\prime }-A_{1}/A_{0}}{{A_{1}}^{\prime }/{A_{0}}^{\prime }}\times 100\%$$

where *X*_MB_% is the single-pass conversion of methyl bromide, *A*_1_*′*, *A*_0_*′*, *A*_1_, and *A*_0_ are the peak areas of CH_3_Br and N_2_ before and after the reaction determined by GC, respectively.

The selectivity of products in groups (*S*_*i*_%) was calculated by the Equation 2:

(2) $$S_{i}\%=\frac{\sum A_{i}\cdot f_{i}}{\sum A_{j}\cdot f_{j}}\times 100\%$$

where *S*_*i*_% is the products' selectivity in a group, and *A*_*i*_ and *f*_*i*_ are the peak areas of products in all carbon numbers' range in target groups and corresponding correction factors on TCD. *A*_*j*_ and *f*_*j*_ are peak areas of all products and corresponding correction factors on TCD.

## Results and discussion

### General characterization of the as-prepared catalysts

**Figure 2:**
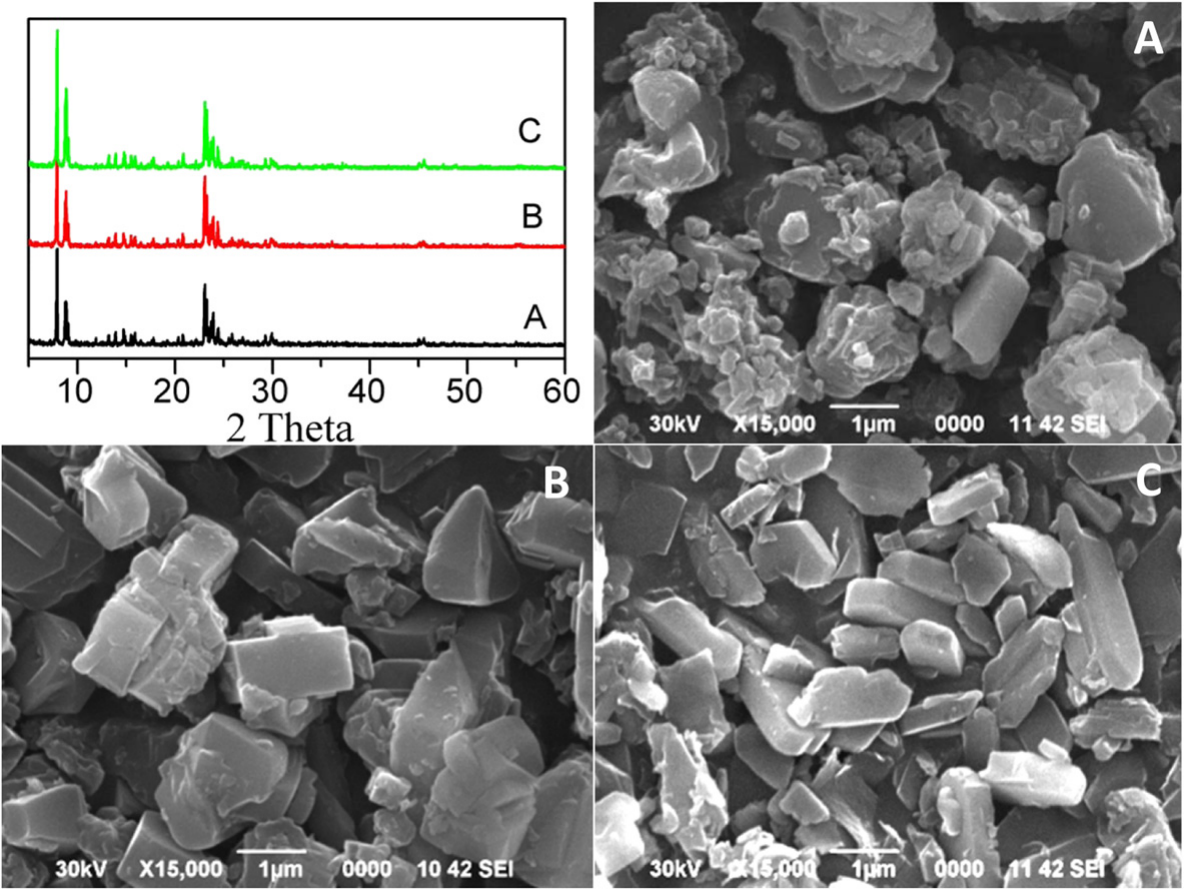
**Powder XRD patterns and SEM images of as-prepared MgHZ catalysts.** Top left, powder XRD patterns; SEM images of as-prepared MgHZ catalysts with Si/Al ratios 50 **(A)**, 100 **(B)**, and 360 **(C)**.

The XRD patterns and SEM images of the as-prepared catalysts with Si/Al ratios of 50, 100 and 360 are presented in Figure 2. The XRD patterns of the three catalysts are well matched with that of HZSM-5 given in the database of standard XRD patterns. The peaks at 7.98, 8.86, and 9.13 ^o^2θ (the last two are partially overlapped) are attributed to the 101, 200 and 111 reflections of typical HZSM-5 phase with MFI structure. All the catalysts showed the HZSM-5 phase which revealed that the preparation process of catalysts did not damage the structure of HZSM-5. Additionally, there is no obvious MgO peak observed in the XRD patterns of these catalysts, which means that MgO species are of low concentration but probably highly dispersed on ZSM-5 support.SEM images of the as-synthesized MgHZ catalysts show that MgHZs with Si/Al ratios of 360, 100, and 50 exhibit fine, aggregated crystals with crystal sizes between 1 and 2 μm. Some fine particles could be observed on the catalyst with low Si/Al ratio of 50 (Figure 2A), which could be due to the poor crystal growth because of much more Al in this composition.

The specific surface areas of the as-prepared catalysts with different Si/Al ratios and parent HZSM-5-360 (HZ-360, Si/Al =360) are listed in Table 1. The BET measurement on the parent HZ-360 showed a surface area of 330.1 m^2^/g and a pore volume of 0.1877 ml/g, while the as-prepared MgHZ-360 showed a surface area of 319.8 m^2^/g and a pore volume of 0.184 ml/g, suggesting that there is only a small decrease in the specific surface area and pore volume during the preparation process.

**Table 1:** BET measurement of as-prepared and spent catalysts

**Catalyst**	**As-prepared**			**Spent**		
	**BET**^**a**^**(m**^**2**^**/g)**	**Volume (ml/g)**	**Pore diameter (nm)**	**BET**^**a**^**(m**^**2**^**/g)**	**Volume (ml/g)**	**Pore diameter (nm)**
MgHZ-50	298	0.1902	0.813	44.5	0.0512	0.811
MgHZ-100	307.8	0.1737	0.729	8.1	0.0213	0.938
MgHZ-360	319.8	0.184	0.674	169.8	0.1048	0.112
MgHZ-360-a^b^	-	-	-	166.04	0.1089	0.68
HZ-360^c^	330.1	0.1877	0.715	-	-	-

^a^Specific surface area; ^b^active catalyst after 30-h testing; ^c^parent HZSM-5 with Si/Al ratio of 360.

### Acidity properties of the as-prepared catalysts

**Figure 3:**
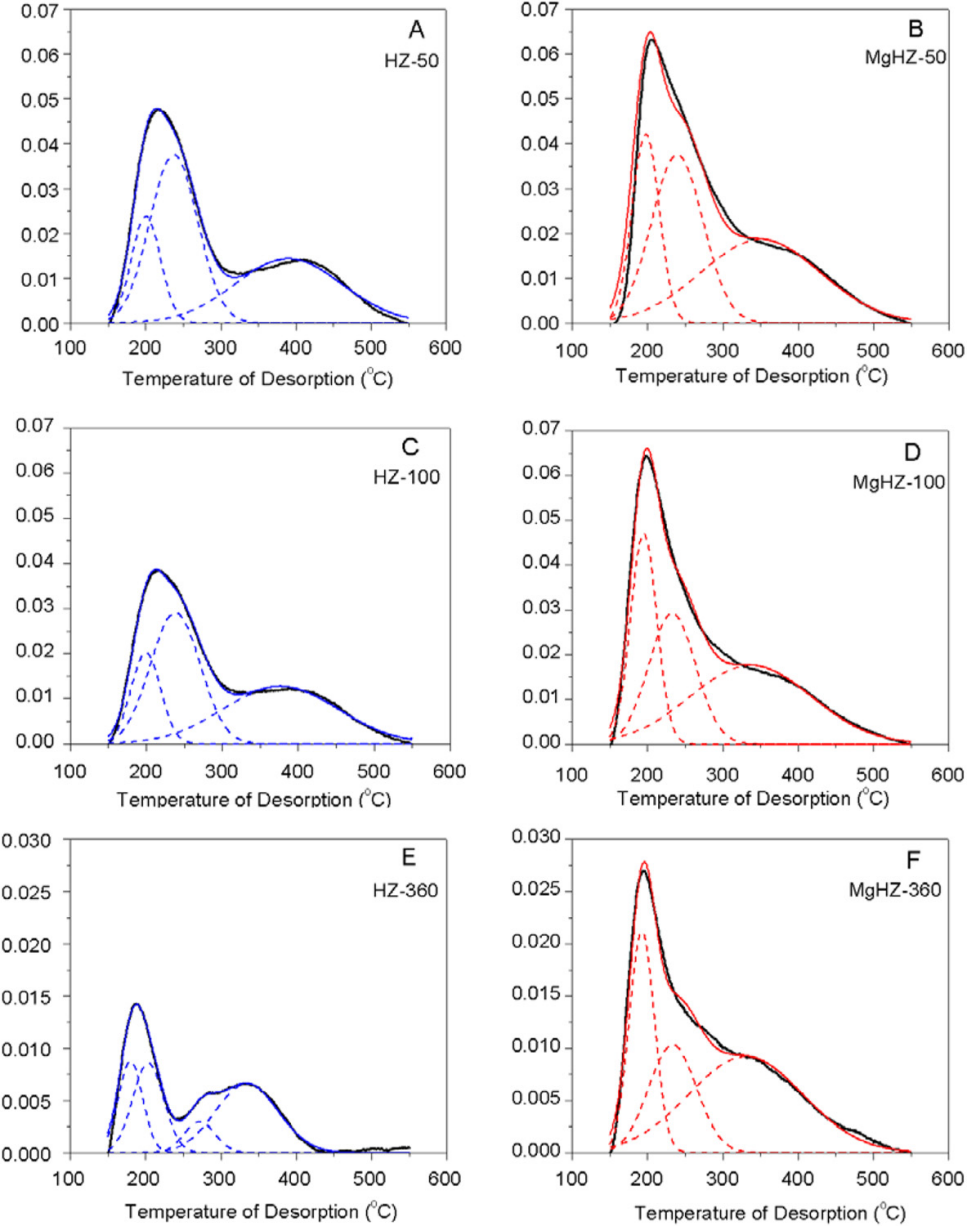
**NH**_**3**_**-TPD profiles of HZs and MgHZs with different Si/Al ratios.** Parent HZs **(A)**, **(C)**, and **(E)** and MgHZ catalysts **(B)**, **(D)**, and **(F)** with Si/Al ratios of 50, 100, and 360. The deconvoluted peaks are shown as dashed lines.

NH_3_-TPD was done to investigate the overall nature and distribution of the acid sites, and the results are presented in Figure 3. According to the asymmetry of the desorption peak, multiple desorption peaks appear to be overlapped. The curves were deconvoluted into individual peaks by Gaussian deconvolution method, which are shown as dashed lines in Figure 3 as well. According to the traditional classification, the deconvoluted peaks could be classified into two kinds of acid strength corresponding to NH_3_-eluted temperature, which are named as weak and strong acid sites, respectively. The quantitative calculations of acid sites with different strengths were estimated from the area of deconvoluted peaks, and the results are listed in Table 2.

**Table 2:** Quantitative calculation of acid sites of parent HZSM-5 and MgHZ catalyst

	**HZ-50**				**HZ-100**				**HZ-360**				
**PT**	**α**	**β**	**γ**	**Total**	**α**	**β**	**γ**	**Total**	**-**	**α**	**β**	**γ**	**Total**
*T*_Des_	200	238	388	-	200	238	375	-	181	201	272	334	-
*C*_Acid_	0.12	0.31	0.26	0.69	0.1	0.25	0.25	0.59	0.04	0.05	0.02	0.07	0.14^a^
%_Int_	17.28	44.98	37.74	100	17.09	41.34	41.56	100	21.17	27.07	9.48	42.29	100
	MgHZ-50				MgHZ-100				MgHZ-360				
PT	α	β	γ	Total	α	β	γ	Total	-	α	β	γ	Total
*T*_Des_	198	240	350	-	195	233	338	-	-	193	234	331	-
*C*_Acid_	0.2	0.33	0.38	0.91	0.21	0.26	0.35	0.82	-	0.09	0.08	0.18	0.36
%_Int_	21.62	36.61	41.76	100	25.3	31.33	43.37	100	-	25.72	23.8	50.47	100

PT, peak type; *T*_Des_, desorbed temperature (°C); *C*_Acid_, acid intensity (mmol/g); %_Int_, peak intensity fraction. ^a^Physical adsorption peak is not taken into account.

The spectra of parent HZSM-5 with Si/Al ratios of 50 and 100 (HZ-50 and HZ-100) contain two peaks centered at temperatures of 200°C and 238°C (here named as peaks α and β, respectively), which are normally referred to as weak acid sites [27]. In the range of strong acid sites from 250°C to 550°C, a broad peak around 380°C is observed (here named as peak γ). However, different locations of weak acid sites were observed for HZ-360 centered at the temperatures of 181°C and 201°C. Comparing the desorption temperature of HZ-360 with those of HZ-100 and HZ-50, it is concluded that the peak around 201°C in HZ-360 could be assigned to peak α and the weaker peak around 181°C (here named as peak -), which probably comes from desorption of physically adsorbed NH_3_. The physical adsorption peak becomes dominant and detectable because of the lower intensity of weak acid sites when ultralow amount of Al was incorporated. Additionally, there is an obvious overlapping of peaks in strong acid range for HZ-360, which could be deconvoluted into two peaks centered at 272°C and 334°C. The reduction of aluminum incorporation, i.e., increasing Si/Al ratio from 50 to 360, could enhance the strength of weak acid β from 238°C to strong acid range of 272°C while reducing the strength of strong acid peak γ from 380°C to 334°C.

After impregnating MgO on HZSM-5, both the intensity and strength of the acid sites of catalysts significantly changed. For MgHZ-360 (Figure 3F), the weakest chemical adsorbed peak, peak α, which is normally assigned to Lewis acid site [28], decreased from 201°C to 193°C. Secondly, the strong acid peak β at 272°C disappeared while a new peak appeared in the range of weak acid sites at 234°C. Lastly, the strongest acid site, which is normally referred to as Brønsted acid site [28], also shifts slightly to the lower temperature from 334°C to 331°C. All the results seem to indicate that the introduction of MgO could reduce the strength of acid sites. However, the intensities of all the peaks in MgHZ-360 were significantly higher than that in parent HZ-360 (Table 2), which means that the introduction of MgO could lead to the generation of extra acid sites. An analogous phenomenon could also be obtained on MgHZ-50 (Figure 3B) and MgHZ-100 (Figure 3D) but with more significant temperature down-side shift and similar intensity increase of approximately 0.22 mmol/g. All the results indicate that the introduction of MgO led to an intensity increment and strength reduction of both strong and weak acid sites.

**Figure 4:**
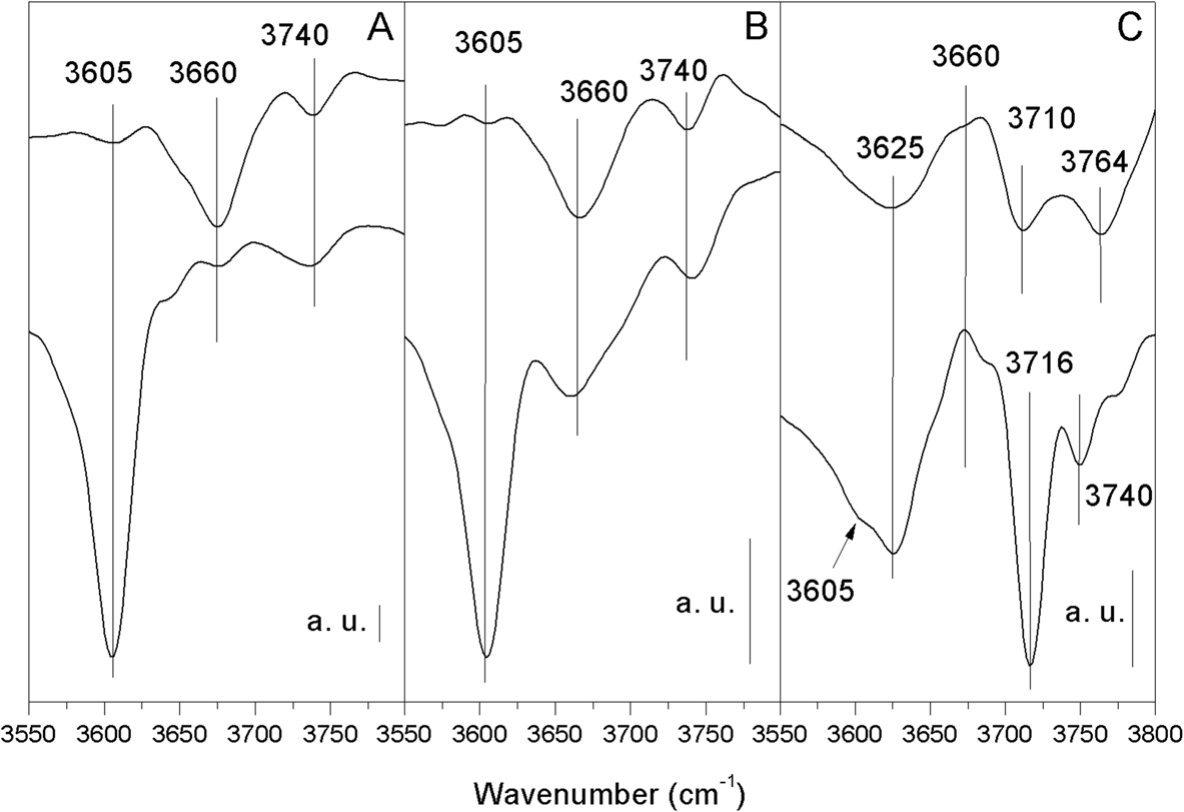
**FTIR spectra of HZs and MgHZs with different Si/Al ratios.** Parent HZs (bottom) and as-prepared MgHZs (above) with Si/Al ratios 50 **(A)**, 100 **(B)**, and 360 **(C)**.

**Figure 5:**
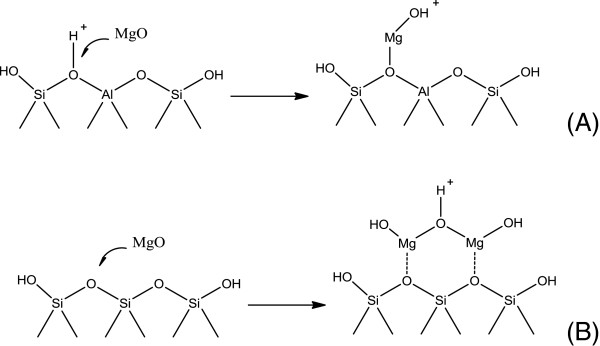
Two types of MgO interaction (A, B) with acidic bridging hydroxyl groups.

FTIR analysis on parent HZs and as-prepared MgHZs was evaluated and the results are presented in Figure 4. As for the HZs with lower Si/Al ratios of 50 and 100, both spectra exhibit a very sharp band around 3,605 cm^-1^ and a small band around 3,740 cm^-1^, which are well known and are assigned to acidic bridging Si-OH-Al hydroxyl groups and terminal Si-OH groups on the zeolite's surface, respectively [28]. After impregnation by MgO, the band located at 3,605 cm^-1^ almost disappeared while the band at 3,740 cm^-1^ barely changed, which indicate that MgO preferentially interacts with the acidic bridging hydroxyl groups (Figure 5A). Additionally, a new band appeared at 3,660 cm^-1^, which is most likely contributed by the interaction between MgO and acidic bridging hydroxyl group. It seems to reveal that a substitution interaction between Mg^2+^ and protons of Si-OH-Al groups to form Mg(OH)^+^ has occurred, which resulted in a decrease of the number of bridged hydroxyl acid sites as reflected in the disappearance of the band at 3,605 cm^-1^ and an increase in the number of the Mg(OH)^+^ sites as reflected in the appearance of a band at 3,660 cm^-1^.

As for HZ with the highest Si/Al ratio of 360, remarkable differences in infrared (IR) bands were observed compared to the other HZs with lower Si/Al ratios. A new sharp band appeared at 3,716 cm^-1^, which can be probably assigned to the asymmetric hydroxyl-bonded silanols as in the case of high-silica zeolite [29]. After impregnating by MgO, two new bands around 3,764 and 3,710 cm^-1^ appeared instead of asymmetric band of silanols at 3,716 cm^-1^. The unique FTIR features of MgHZs with different Si/Al ratios indicate a different interactional mechanism between MgO and HZs surface. Because of the low amount of alumina incorporation in HZ-360, fewer Si-OH-Al hydroxyl groups are formed. The bands at 3,764 and 3,710 cm^-1^ could probably be derived from the surface OH groups of Mg-OH (Figure 5B, 3,764 cm^-1^ for surface OH groups directly attached to the Mg^2+^ cations and 3,710 cm^-1^ for interstitial OH groups shared between two adjacent Mg^2+^cations). Compared to the crystalline MgO [30], it should be noted that these bands shifted to lower wavenumbers from 3,570 and 3729 cm^-1^, which is probably related to a monolayer of a different MgO phase crystallized at the interface of HZ support.

**Figure 6:**
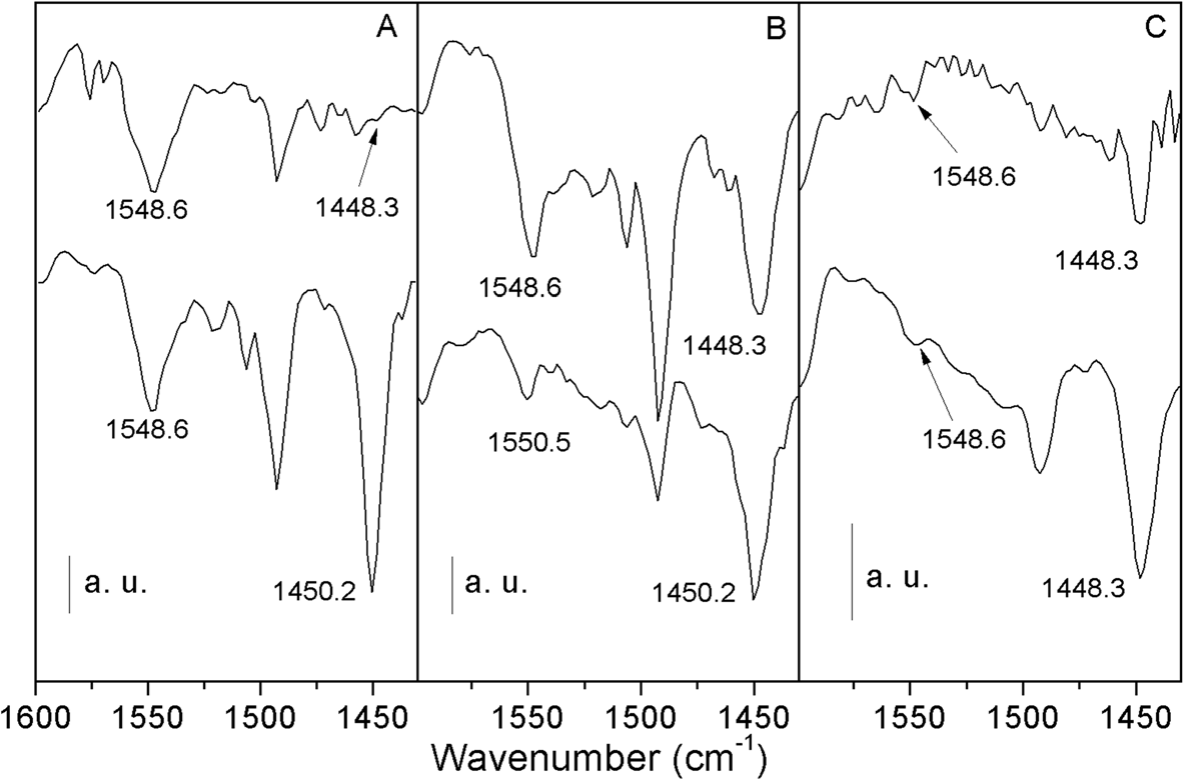
**FTIR spectra after pyridine adsorption on HZs and MgHZs with Si/Al ratios.** Parent HZs (above) and as-prepared MgHZs (bottom) with Si/Al ratios 50 **(A)**, 100 **(B)**, and 360 **(C)**.

The adsorption of pyridine on solid catalyst materials and its analysis by FTIR have been widely used to distinguish the presence of Lewis or Brønsted acid sites. Herein, the acid type was identified by collecting IR spectra from pyridine adsorption on HZs or MgHZs (Figure 6). The spectra of pyridine adsorbed on parent HZs exhibit characteristic bands at 1548 and 1448 cm^-1^, which are attributed to Brønsted acid sites and Lewis acid sites, respectively. The band at 1490 cm^-1^ is assigned to pyridine on both Brønsted and Lewis acid sites. The spectrum from HZ with low Si/Al ratio of 50 only exhibits an intense band at 1548 cm^-1^, which indicates that most of the acid sites are Brønsted acid type. In contrast, HZ with highest Si/Al ratio of 360 only exhibits an intense band at 1448 cm^-1^, which suggests that most of the acid sites are Lewis acid type.

After the impregnation of MgO, noticeable changes of the IR spectra are observed. When MgO was impregnated on HZ-50 and HZ-100, the intensity of the band representing Brønsted acid sites decreased while a significant increase of the band representing Lewis acid sites occurred. In addition, the band representing Lewis acid site at 1,448 cm^-1^ shifted to 1,450 cm^-1^, which could be explained by the transformation of bridged hydroxyl groups to Mg(OH)^+^ groups. As for HZ-360, a dramatic increase in Lewis acid sites occurred along with a slight increase of Brønsted acid sites. Besides the existence of Lewis acid sites in MgHZ-360, which are mostly generated by charge imbalance of the MgO framework, the presence of Brønsted acid sites suggests that they may have been generated by interstitial OH groups shared between two adjacent Mg^2+^ cations.

### Catalytic performance and stability test

**Figure 7:**
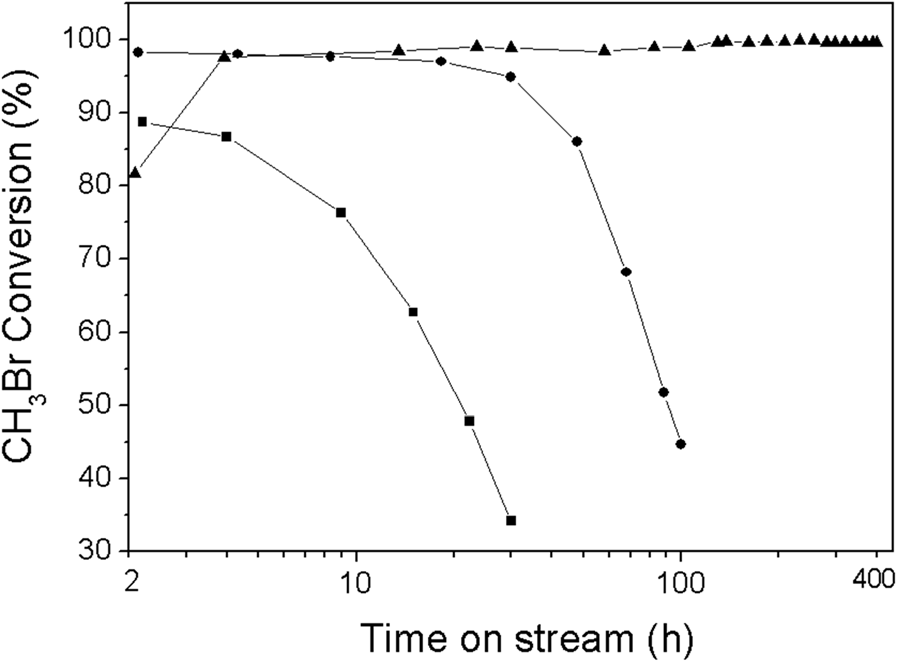
**CH**_**3**_**Br conversion with time on stream.** MgHZs with Si/Al ratios of 360 (▲), 100 (●), and 50 (■) (WHSV =0.5725 h^-1^; reaction temperature =280°C).

In order to investigate the acidity influence on the CH_3_Br dehydrobromination reaction, MgHZ catalysts were evaluated and the results of catalytic stability are depicted in Figure 7. It can be seen that catalysts showed totally different performances even at the initial period of reaction, i.e., 88.7%, 98.2%, and 81.7% of CH_3_Br conversions were obtained for MgHZ-50, MgHZ-100, and MgHZ-360, respectively. Furthermore, the catalysts also showed differences in the stability of their performance. As for MgHZ-100, the deactivation of the reaction was pronounced and led to a decrease of CH_3_Br conversion from 98.2% to 44.7% in 100 h. Almost the same trend of CH_3_Br conversion was observed over MgHZ-50 leading to a decrease in conversion from 88.7% to 34.2% in just 30 h. However, the MgHZ-360 catalyst prepared from HZ with highest Si/Al ratio of 360 was found to be the best, as the CH_3_Br conversion reaction was maintained above 99.0% during 400 h of online test (Figure 7).

It is interesting to note that an induction period was observed on MgHZ-360 when CH_3_Br conversion increased from 81.7% to 97.5% during the initial period of 4 h. After that, CH_3_Br conversion slightly increased to 99% in 100 h on the stream and maintained this level in the next 300 h. This phenomenon is in line with the observation of the induction period in the MTG process [31,32] or dehydrochlorination process [8,33], which is mainly due to the active ‘carbon pool’ molecular formation form methyl reactant. Actually, among the investigations of the reaction mechanisms of MTH, some authors also mentioned that the dehydrohalition of methyl halides to hydrocarbons might have a similar reaction mechanism to the MTH reaction [4]. Compared with methanol or CH_3_Cl as reactant, the dehydrobromination of CH_3_Br had a prolonged induction period to facilitate a direct observation. The slow kinetics is possibly related to the relative lower intensity of acid sites on MgHZ-360.

**Figure 8:**
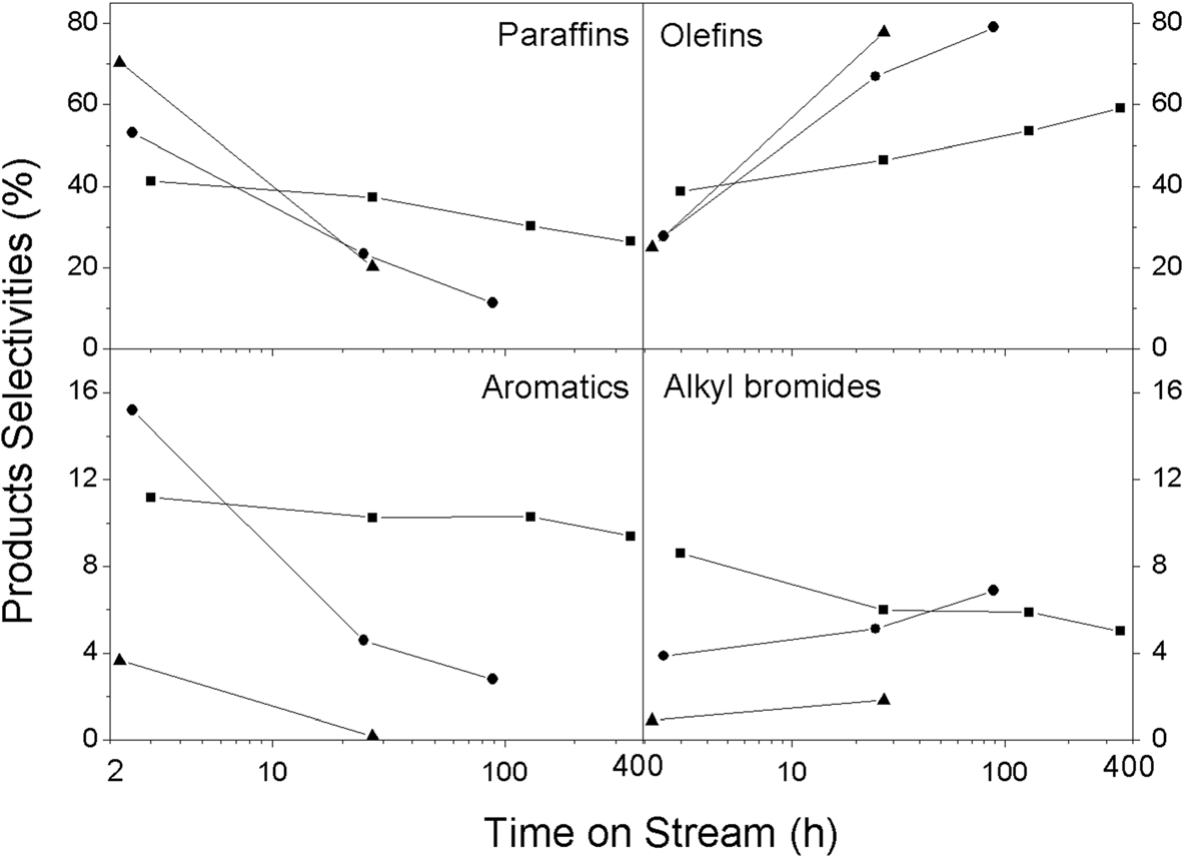
**Products distribution with time on stream over MgHZs with different Si/Al ratios.** MgHZs with Si/Al ratios of 360 (▲), 100 (●), and 50 (■).

The products of CH_3_Br dehydrobromination reaction were analyzed by GC/MS. The results revealed that the products of all the three catalysts were complicated, which included paraffins, olefins, aromatics, and fewer alkyl monobromides in a wide range of carbon numbers from C_1_ to C_11_ (data is not shown here). It is easy to understand that the complexity of the dehydrobromination reaction leads to complex product distribution because of the carbon pool mechanism. The variations of product selectivity of the three catalysts as a function of time on stream are presented as Figure 8, which shows that paraffins and olefins were the main products. However, remarkable differences of product distribution over MgHZs are also observed. When MgHZ-360 was used as a catalyst, only 41.4% selectivity of paraffins was obtained at the initial period, while 53.2% and 70% selectivities of paraffins were obtained with MgHZ-100 and MgHZ-50, respectively. In contrast to the selectivity of paraffins, the selectivity of olefins showed the opposite trend, which led to 25.1%, 27.8%, and 38.9% selectivities over MgHZ-50, MgHZ-100, and MgHZ-360 catalysts, respectively. As concluded in acidity property characterization, lower intensity and weaker acid sites with Lewis acid character were formed on MgO-modified HZSM-5 catalyst with higher Si/Al ratio, herein combining with catalytic results, which indicate that lower acid intensity and higher Lewis acid concentration prefer to form alkenyl products via primary splitting reaction from carbon pool molecular. On the contrary, the catalyst with higher acid intensity and higher Brønsted acid concentration preferably forms paraffins and aromatics, which could be explained by the enhancement of secondary reactions from olefins to hydrogen-rich paraffins and hydrogen-poor aromatics even at the initial period of the reaction.

However, the selectivity to paraffins and aromatics decreased sharply with time on stream with a sharp increase of selectivity for olefins. When reaction was carried out over MgHZ-50, the selectivity of paraffins decreased from 70.4% to 20.2% rapidly within 27 h while selectivity of olefins increased from 25.1% to 77.8%. Analogously, the sharp decreases in selectivity of paraffins and aromatics were accompanied by sharp increases of selectivity for olefins over MgHZ-100 during the first 25 h of reaction. A minimal decline in selectivity was obtained over MgHZ-360, even when the reaction was tested for the longest time of 400 h. Combining catalytic performance test results and the abovementioned acid properties of catalysts, it could be concluded that catalysts with higher acid intensity and Brønsted acid character tend to form large amounts of paraffins and aromatics, which could be responsible for the quick deactivation behavior as well.

### Coke characterization of the spent catalysts

**Figure 9:**
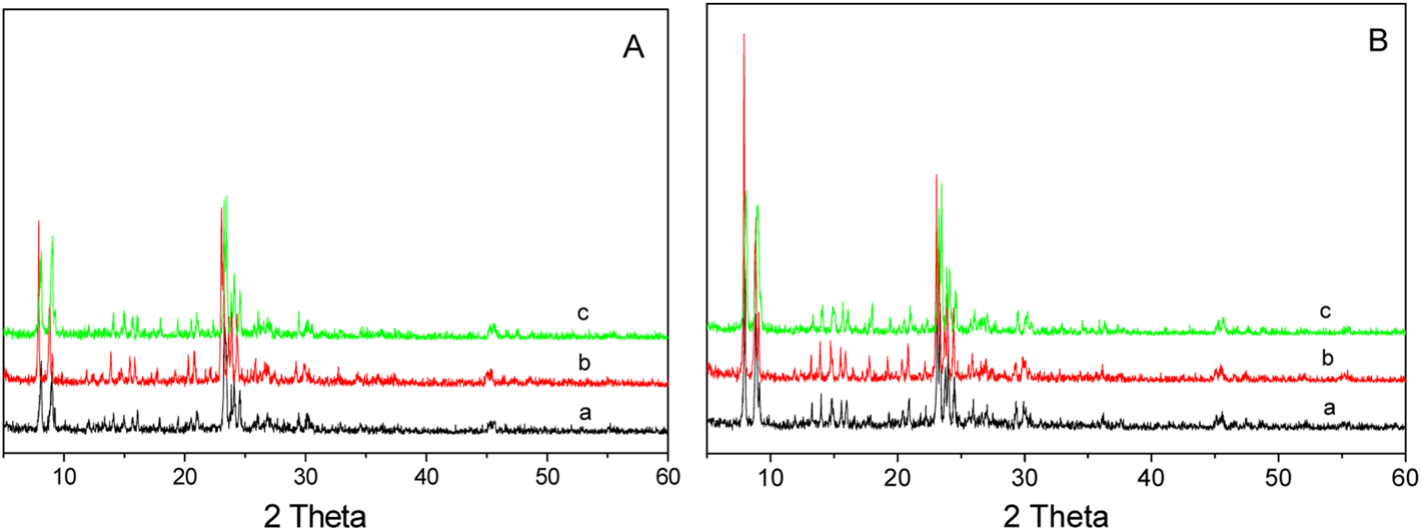
**Powder XRD patterns of spent (A) and regenerated catalysts (B).** a, MgHZ-50; b, MgHZ-100; c, MgHZ-360.

The XRD patterns of discharged catalysts after reaction were identified and illustrated in Figure 9A. Compared with fresh catalysts, the typical diffraction peaks at 7.98°, 8.86°, and 9.13° 2*θ* were remarkably reduced, which indicated that the surface crystal might be somewhat destroyed or covered by coke deposit. Another XRD measurement was also conducted over the regenerated catalysts by calcining samples under air flow for 4 h at 450°C to remove the possible coke deposit. As shown in Figure 9B, the patterns of the regenerated catalysts were totally recovered and similar to the fresh ones, which indicated that the weakened signals on the spent catalysts are mostly due to the coke deposits on the surface of the crystal.

The BET measurements of the discharged catalysts after reaction are shown in Table 1 as well. The results show that the specific surface area of spent catalyst with lower Si/Al ratio decreased sharply, especially for the catalyst with Si/Al ratio of 100 which drastically decreased from 307.8 to 8.1 m^2^/g although it was tested for a shorter time. The catalyst with highest Si/Al ratio of 360, which had the best catalytic performance, showed a much smaller decrease in surface area, i.e., from 319.8 to 169.8 m^2^/g. The BET measurement of MgHZ-360 catalyst which was tested for 30 h on stream with up to 99% CH_3_Br conversion (denoted as MgHZ-360-a) showed a decline of its specific surface area to 166.04 m^2^/g. This decrease in the surface area is almost equal to the decline of the specific surface area to 169.8 m^2^/g for the catalyst tested for long term, which seems to suggest that the coke deposit was formed during the initial period of the reaction.

**Figure 10:**
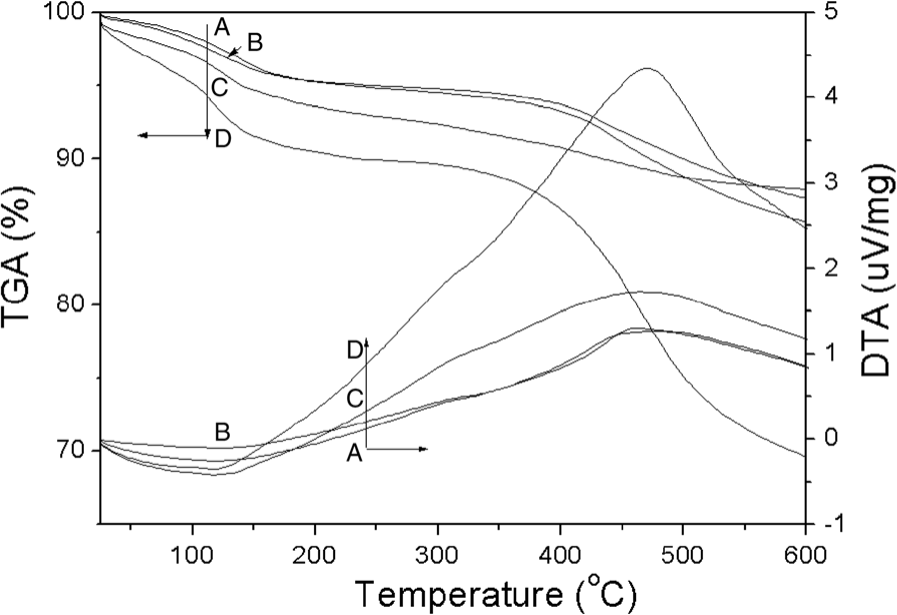
**TGA/DTA of spent and activated catalysts.** Spent MgHZ-50 **(A)**, MgHZ-100 **(B)**, and MgHZ-360 **(D)**, as well as activated MgHZ-360-a catalyst which is tested for 30 h **(C)**.

TGA/DTA (Figure 10) shows two obvious weight losses for all the spent catalysts in the temperature range of 25°C to 600°C. The first weight loss with endothermic effect (DTA curve) in the range of 25°C to 200°C is attributed to water desorption, and the second weight loss in the range of 350°C to 600°C, accompanied by exothermic effect, is assigned to the coke removal. The amounts of coke deposit calculated from TG curves are listed in Table 3. It is interesting to note that although the specific surface areas of catalysts with lower Si/Al ratios decreased sharply, the coke amounts were only 8.2% and 10.5% for MgHZ-50 and MgHZ-100, respectively. In contrast, the coke amount on catalyst with highest Si/Al ratio of 360 was 23.1% accompanied by minimal specific surface area decline after the longest catalytic lifetime.

**Table 3:** Calculated results of spent and active catalysts from TGA/DTA

	**Catalysts**			
	**MgHZ-50**	**MgHZ-100**	**MgHZ-360**	**MgHZ-360-a**^**a**^
Testing time (h)	30	100	400	30
Coke content (wt.%)	8.2	10.5	23.1	4.8

Coke content was calculated according to the weight loss of TG result in the range of 300°C to 700°C. ^a^Activated MgHZ-360-a catalyst which is tested for 30 h.

From the shape of the DTA curves, we can see that acute exothermic peaks were obtained during the coke combustion over all the spent catalysts (Figure 10A, B, D), while a somewhat gently sloping exothermic peak was obtained over activated MgHZ-360-a (Figure 10C). This slight difference in slope seems to indicate that a different coke type was formed on MgHZ-360-a during the reaction. In fact, after the catalyst MgHZ-360 was tested for 30 h on stream, the dehydrobromination reaction just finished its induction period and entered the steady stage. It can be postulated that the weight loss of MgHZ-360-a in the thermal treatment process was not derived from the removal of the coke deposit but from the carbon pool species. The calculated weight losses of spent and activated MgHZ-360 catalysts support the above conclusion. When MgHZ-360 catalyst was tested for the period of 30 h, only a slight amount of weight loss, i.e., 4.8% was obtained. After prolonging the testing time to 400 h, a sharp increase of coke deposit with the value of 23.1% (Table 3) was obtained for MgHZ-360 with a CH_3_Br conversion of up to 99%.

It is well known that coke could be formed on both external and internal surfaces of the catalyst and it could be affected by various parameters [34]. Internal coke formation may cover certain acid sites causing their deactivation, while external coke formation is more complicated which could cause hindrance to diffusion or block the exits of the channels of the catalyst. When the reaction was carried out over the catalyst with different acid intensity and type, the discrepancy between sharply decreased specific surface area and minimal coke deposit on MgHZ-50 and MgHZ-100 can be explained by the formation of coke deposit mainly on the external surface, which blocked the diffusion of reactant. On the other hand, the larger amount of coke deposit and relatively lower decline of specific surface area of MgHZ-360 seems to indicate a different location for the coke deposit, i.e., mainly in the channels of catalyst. The coke deposit apparently covered only a certain number of acid sites leaving enough reactive sites for the conversion of CH_3_Br. The minimal amount of coke deposit and almost the same specific surface area decline on MgHZ-360-a (Table 1) also confirm this conclusion.

**Figure 11:**
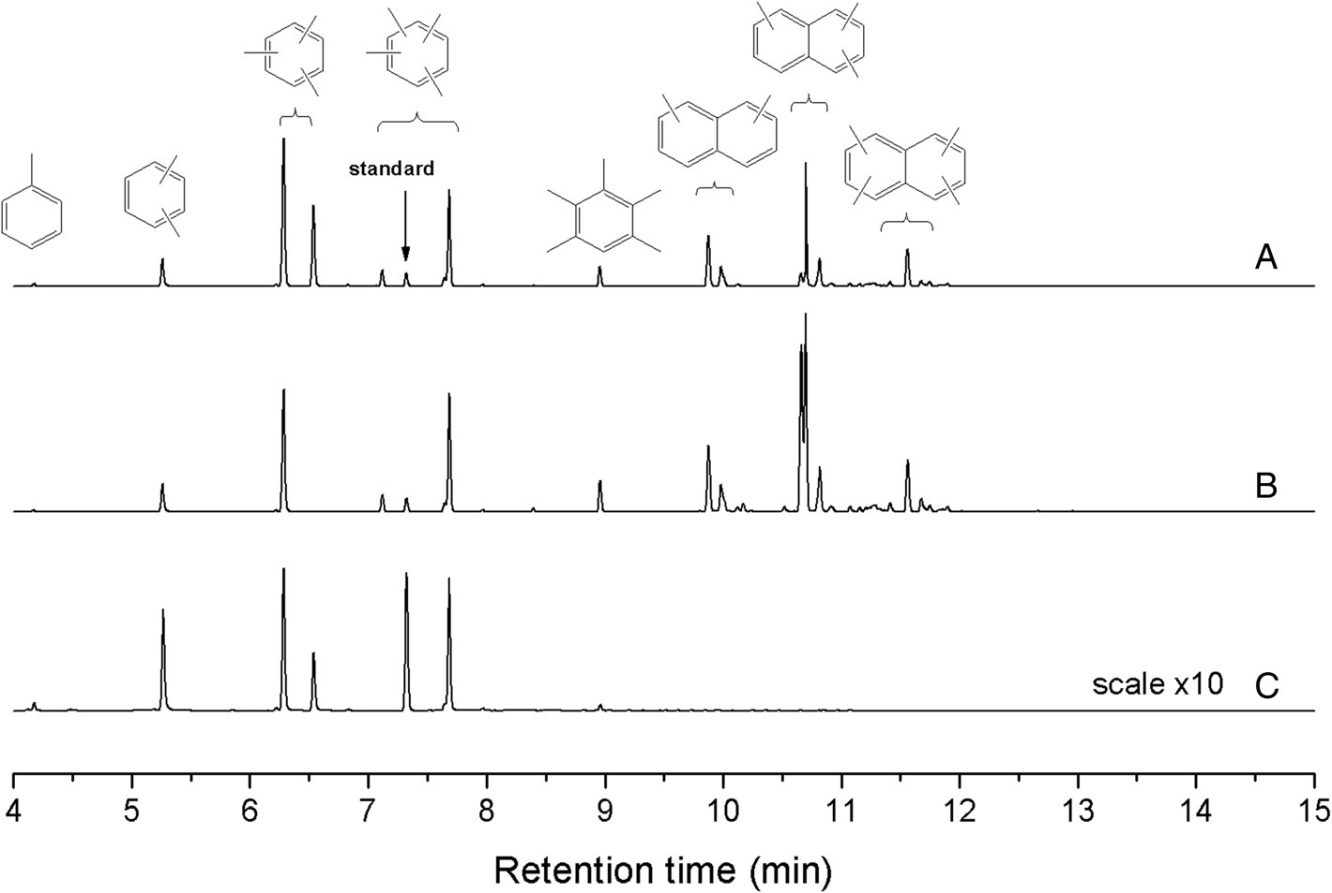
**GC/MS analysis of trapped organic compounds in different catalysts.** Spent catalysts of MgHZ-50 **(A)**, MgHZ-100 **(B)**, and MgHZ-360 **(C)**.

Figure 11 presents the GC/MS result of the organic compounds trapped in the spent catalyst. Compared to the reaction of methanol to hydrocarbon (MTH) process, aromatics were detected in extracted organic phase but with some differences. Methylated naphthalene compounds were observed in the spent catalysts with lower Si/Al ratio, which is in contrast to the results of Bjørgen et al. [35]. They found that no methylated naphthalene was trapped in the HZSM-5 when using methanol as the reactant. Organics formed in the MgHZs depended on the acid intensity and type to a certain extent (Figure 11), which means that the catalyst with higher acid site intensity and more Brønsted acid type tended to generate naphthalene species while only methyl substituted benzene species were observed in the retained catalyst with higher acid site intensity and more Lewis acid type.

As is well known, the pentasil-type ZSM-5 has been the candidate among the medium-pore zeolites to be investigated as catalyst for the MTH conversion reaction [4]. The pore-size of MgHZ is a perfect fit for methyl benzene, which confirms that the methyl naphthalene species was mainly formed outside the catalyst and blocked the diffusion of reactant into the channels and thus causing the deactivation. Another finding is that part of the coke deposit retained in spent MgHZ-360 is insoluble in both organic and aqueous phases. This indicates that coke of graphitic character may have formed. These phenomena explain the discrepancy between the total coke content determined by TG and MS of the trapped organics.

## Conclusions

Three kinds of catalysts were prepared by loading 2.0 wt.% MgO on HZSM-5 zeolites with different Si/Al ratios to investigate the effect of acid strength and intensity on methyl bromide dehydrobromination. The structure and crystallinity of HZSM-5 were retained after impregnation with Mg salt, but no Mg phases could be detected by XRD. The strength and intensity of strong acid sites of the catalyst were attenuated while the weak acid sites were enhanced, which may be due to Mg(OH)^+^ formed from MgO on HZSM-5 with low Si/Al ratio or charge imbalance of the MgO framework on the surface of HZSM-5 with high Si/Al ratio. Py-FTIR confirmed that Lewis acid sites were highly enhanced by MgO impregnation. Methyl bromide dehydrobromination test revealed that both the activity and lifetime of catalyst are highly affected by catalyst acidity. The selectivity to paraffins and aromatics was increased on catalysts with high acid intensity and more Brønsted acid concentration because of the high secondary hydrogen transfer reaction, which could be responsible for the quick deactivation behavior as well. TGA/DTA and GC/MS analysis revealed that polymethylated naphthalenes species were formed on stronger acid sites during the initial period, while graphitic carbon was formed on the weaker sites during the stable stage.

## Competing interests

The authors declare that they have no competing interests.

## Authors' contributions

ZL and XZ generated the research idea and designed the experiments. ZL and ZZ carried out the experiments and drafted the manuscript. SK and XZ revised the manuscript. WX, SK, ZY, and XG participated in the analysis of data. XZ supervised the whole work and given final approval of the version to be published. All authors read and approved the final manuscript.
